# sEMG-controlled forearm bracelet and serious game-based rehabilitation for training manual dexterity in people with multiple sclerosis: a randomised controlled trial

**DOI:** 10.1186/s12984-023-01233-5

**Published:** 2023-08-19

**Authors:** Selena Marcos-Antón, Alberto Jardón-Huete, Edwin Daniel Oña-Simbaña, Aitor Blázquez-Fernández, Lidia Martínez-Rolando, Roberto Cano-de-la-Cuerda

**Affiliations:** 1 Faculty of Health Sciences, International PhD School, Rey Juan Carlos University, 28008 Madrid, Spain; 2 Department of Physical Therapy, Occupational Therapy, Physical Medicine and Rehabilitation, Faculty of Health Sciences, Rey Juan Carlos University, 28922 Alcorcón, Madrid Spain; 3 Asociación de Leganés de Esclerosis Múltiple (ALEM), 28915 Leganés, Madrid Spain; 4 Robotics Lab, Department of Systems Engineering and Automation, University Carlos III of Madrid, 28911 Leganés, Madrid Spain; 5 Rey Juan Carlos University Hospital of Móstoles, 28933 Madrid, Spain

**Keywords:** Multiple sclerosis, MYO Armband, Rehabilitation, Serious games, Strength, Upper limb, Virtual reality

## Abstract

**Background:**

Muscle strength and dexterity impairments are common among patients with multiple sclerosis (MS) producing limitations in activities of daily living related to the upper limb (UL). This study aimed to evaluate the effectiveness of serious games specifically developed for the MYO Armband® capture sensor in improving forearm and wrist mobility, UL muscle strength, dexterity, fatigue, functionality, quality of life, satisfaction, adverse effects and compliance.

**Methods:**

A double-blinded (allocation concealment was performed by a blinded investigator and by blinding for assessors) randomised controlled trial was conducted. The sample was randomised into two groups: an experimental group that received treatment based on UL serious games designed by the research team and controlled by the MYO Armband® gesture capture sensor, along with conventional rehabilitation and a control group that received the same conventional rehabilitation for the UL. Both groups received two 60-min sessions per week over an eight-week period. Wrist range of motion (goniometry), grip muscle strength (Jamar® dynamometer), coordination and gross UL dexterity (Box and Block Test), fatigue (Fatigue Severity Scale), functionality (ABILHAND), quality of life (Multiple Sclerosis Impact Scale-29), adverse effects (Simulator Sickness Questionnaire, SSQ), perceived workload (NASA-Task load index), satisfaction (Client Satisfaction Questionnaire-8 (CSQ-8), Satisfaction with Technology Scale, System Usability Scale (SUS) and QUEST 2.0) and compliance (attendance) were assessed in both groups pre-treatment, post-treatment and during a follow-up period of 2 weeks without receiving any treatment.

**Results:**

Significant differences were observed in the experimental group compared to the control group in the assessment of forearm supination (p = .004) and grip strength (p = .004). Adverse effects were minimal (SSQ: 7/100 points) and perceived workload was low (NASA-Task Load Index: 25/100 points) in the experimental group. The MYO Armband® technology proved to be useful for the participants (SUS: 80.66/100) and the satisfaction scales received high scores (QUEST 2.0: 59.4/70 points; Satisfaction with Technology: 84.36/100 points). There were significant differences between the groups in terms of attendance percentage (p = .029).

**Conclusions:**

An experimental protocol using MYO Armband®-based serious games designed for UL rehabilitation showed improvements in active wrist range of motion and handgrip strength in patients with MS, with high satisfaction, minimal adverse effects and workload and excellent compliance.

*Trial registration number:* This randomised controlled trial has been registered at ClinicalTrials.gov Identifier: NCT04171908.

## Introduction

Multiple sclerosis (MS) is a chronic inflammatory demyelinating disease that affects the Central Nervous System (CNS) [1, 2]. The pathologic hallmark of MS consists of focal demyelinated plaques within the CNS, with variable degrees of inflammation, gliosis, and neurodegeneration [3]. These alterations are linked to axono-neuronal loss and problems in nerve conduction, resulting in slowed and/or blocked signals, causing characteristic symptoms of this disease [3, 4]. MS is the most common neurological condition leading to disability in young adults in Europe and North America. Currently, its aetiology is unknown and is believed to have a possible multifactorial origin [4].

MS is characterised by a wide range of symptoms and progression patterns, which significantly impact the quality of life of affected individuals. Specifically, upper limb (UL) impairments have a high prevalence, influencing functionality, independence and quality of life [5]. This issue is present in over 60% of individuals at the time of diagnosis and happens in a greater extent from the beginning of the course of the disease [6]. Pisa et al. [7] highlight that patients suffering from MS report problems with sensitivity, strength, fine manual dexterity and gross motor skills in the UL. As a result of these impairments, negative effects on employability occur, leading to adverse changes in their economy, health and social life [6].

Although clinical and functional impairments in the lower limbs have been extensively studied in patients with MS, the UL are also frequently affected [5]. Johansson et al. [8] observed that, out of 219 people with MS, 76% of patients showed UL impairments, with 50% of them experiencing moderate impairments. Lamers et al. [9] indicated that strength is the primary variable for performing activities of daily living (ADLs), while active range of wrist dorsiflexion and thumb sensitivity are associated with the ability to perform functional tasks. Cattaneo et al. [10] found that manual dexterity is crucial for performing household tasks and that limitations in participating in these tasks are associated with a higher predisposition to developing cognitive deficits. Therefore, several authors emphasise that impairments in UL motor skills are linked to the performance of ADLs, which are also related to functional independence and impact on quality of life in individuals with MS [6, 11].

Despite the emergence of new drugs aimed at modifying the course of the disease, there is currently no curative treatment for MS. Therefore, pharmacological therapy is complemented with rehabilitation treatment to maintain functional capacity and promote adaptation to the changes caused by the progression of MS [12]. However, conventional rehabilitation treatment for people with MS is sometimes referred to as monotonous, which can lead to a loss of motivation and adherence to the treatment [13]. In recent years, new intervention strategies have been introduced, such as virtual reality (VR), which enhance patient motivation through the practice of functional tasks in virtual environments that provide feedback on the results achieved, simulating ADLs. It is also important to note that VR allows the creation of environments to perform tasks that may be difficult to carry out in real life, as well as the playful nature of the activities proposed through these devices, generating an interesting element of competitiveness or challenge that increases the patient's level of motivation [14, 15]. All of this promotes active participation and, consequently, increases adherence to rehabilitation treatment.

One of the devices that can be linked to VR systems is the MYO Armband® surface electromyography (sEMG) and motion capture sensor (Thalmic Labs), which was designed to recognise forearm gestures based on muscle activation. The sensor consists of eight surface electrodes and a 9 degrees of freedom inertial measurement unit (IMU) that includes an accelerometer, magnetometer and gyroscope, each with three degrees of freedom. MYO Armband® has a signal tracking frequency of 200 Hz for sEMG and 50 Hz for the IMU, allowing for three-dimensional motion data collection [16, 17]. The default gestures that this device is capable of detecting are wrist flexion and extension, open hand, handgrip and pinch. These gestures are detected by the sensors and the information is transmitted to a computer through wireless communication via Bluetooth® using a USB-type receiver provided by the manufacturer (it is not possible to connect the device with a generic Bluetooth receiver). In this way, the patient gains the ability to control the device, facilitating its use without the need for other accessories or attachments. It also has a haptic feedback system through vibration and a rechargeable long-lasting lithium-ion battery. This system provides quantitative data on muscle activity that can be used not only as an assessment strategy but also as a semi-immersive VR therapeutic tool through the use of serious games [16–19].

The development of these new VR technologies has provided professionals working in the field of neurological rehabilitation with the opportunity to extend patient care for individuals with MS as a complement to their conventional rehabilitation program, achieving higher treatment intensity and sometimes at a sustainable cost [20]. However, there has been a lack of studies on the effects of VR on manipulative skills in patients with MS and, to the best of our knowledge, there are no high-quality studies investigating the use of the MYO Armband® device as a tool for treating UL impairments in individuals with MS [19].

For the reasons described above, conducting a randomised controlled trial (RCT) to study the effects of the MYO Armband® motion capture device, through specifically designed games, as an adjunct to conventional treatment for individuals with MS is justified.

## Objectives

The aim of this study was to investigate the effects of the MYO Armband® motion capture system, along with specifically designed video games, in combination with a conventional physical therapy program, on active wrist range of motion, grip strength, motor dexterity, fatigue, functionality and quality of life related to UL treatment in patients with MS. As secondary objectives, the study aimed to analyse the occurrence of adverse effects during the treatment, the perceived workload level by the participants, the usefulness of the technological system, satisfaction with the technology used and the service provided, as well as the level of treatment adherence.

Our primary hypothesis was that a structured protocol, using MYO Armband® motion capture system, in combination with a conventional physical therapy program, could enhance active wrist range of motion, grip strength, motor dexterity, fatigue, functionality and quality of life in patients with MS. Our secondary hypothesis was that no side effects would be perceived, as well as a low workload level and high satisfaction and adherence by the experimental group (EG).

## Methodology

### Study design

A double-blind (allocation concealment was performed by a blinded investigator and by blinding for assessors) randomized controlled trial (RCT) (NCT04171908, clinicaltrials.gov) was conducted. Consolidated Standards of Reporting Trials (CONSORT) statement was consulted to help authors improve the reporting of the RCT. A non-probabilistic sampling of consecutive cases was performed. The sample was randomised into two study groups: the EG and the control group (CG), using the QuickCalcs GraphPad® software by a computer-generated sequence. The allocation was performed by a blinded investigator. The EG received a conventional physical therapy program along with the application of a VR protocol and specifically designed video games for UL treatment in individuals with MS. The CG received only the conventional therapy program. All interventions were carried out at the Leganés Association of Multiple Sclerosis (ALEM) in Madrid, Spain.

This protocol was approved by the Ethics and Research Committee of Rey Juan Carlos University with reference number 2310202119821. Written permission was obtained from all selected individuals through the informed consent form.

### Participants

Recruitment process involved formal letters, posting flyers, sending emails, verbal announcements postings to online bulletin boards and social media sites. Once a patient was recruited, a medical doctor checked whether the inclusion and exclusion criteria were met and determined the most affected UL of the patients who met the inclusion criteria in the study.

The study inclusion criteria were as follows: age between 20 and 65 years; confirmed diagnosis of MS according to the McDonald criteria [21], with over two years evolution; Expanded Disability Status Scale (EDSS) score between 3.0 (moderate disability in one functional system, or mild disability in three or four functional systems; a virtually unlimited walking perimeter capacity) and 7.5 (unable to take more than a few steps; restricted to wheelchair and may need aid in transferring; can wheel self but cannot carry on in standard wheelchair for a full day and may require a motorised wheelchair); stable medical treatment for at least six months prior to the intervention; a score of 4 points or less in the "Pyramidal Function" section of the EDSS functional scale; muscle tone in the UL not exceeding 2 points on the Modified Ashworth Scale (moderate hypertonia, increased muscle tone for most of the range of motion, but the affected part can be easily moved passively); muscle strength equal to or greater than 3 points in the UL; absence of cognitive impairment, with the ability to understand instructions and obtain a score of 24 or higher on the Mini-Mental State Examination [22]; and a score of 2 points or less in the "Mental Functions" section of the EDSS.

The exclusion criteria were a diagnosis of another neurological illness or musculoskeletal disorder different to MS; diagnosis of any cardiovascular, respiratory, or metabolic disease, or other conditions that may interfere with the study; suffering a relapse, an exacerbation or hospitalisation within the last 3 months prior to commencement of the assessment protocol or during the therapeutic intervention process; receiving a cycle of intravenous or oral steroids within 6 months prior to the start of the assessment protocol and during the therapeutic intervention period; receiving treatment with botulinum toxin in the 6 months prior to the beginning of the study; presence of uncorrected visual disorders; and a history of photosensitive epilepsy related to the use of video games.

The estimated effect size for the main outcome measure (gross manual dexterity) was 0.25 (medium effect size). A correlation of 0.5 between repeated measures was assumed. With a statistical power of 0.80, an alpha error of 0.05 and a total of 3 measurements taken in two participant groups, a minimum of 28 participants was required, as calculated using G*Power® software. Accounting for a 10% potential loss to follow-up, a sample size of 30 patients (15 subjects per group) was considered for this study.

### Intervention

All groups received the intervention between January 2022 and April 2023. Both the EG and the CG received two 60-min sessions per week over an eight-week period (a total of 16 sessions per group).

The CG received a specific conventional physical therapy intervention by a physical therapist expert in MS patient care. This intervention was based on conventional physical therapy exercises [23, 24], including shoulder, elbow, wrist and fingers joint mobilisation, forearm and hand muscle strengthening exercises, work on gross and fine motor skills and practice of functional tasks aiming to mimic the movements included in the specifically designed games for the intervention of the EG.

The EG received the same conventional physical therapy treatment (45 min) in addition to a semi-immersive VR intervention using the MYO Armband® sensor (15 min) and specifically designed video games for this protocol. The intervention was implemented by an expert therapist proficient in the use of the technology. The patient's starting position was seated in front of a table at mid-trunk height, with the elbow at 90° of flexion and the forearm in a neutral pronation-supination. Manual assistance was provided by the therapist when necessary.

In each video game session, prior to using the device, gesture calibration was performed to improve sampling accuracy and individualise the treatment for each participant. The calibration process included training the system's gesture classifier to optimize the device's performance and minimise the effects resulting from the placement of the armband. It should be noted that the armband is manually placed at an approximate distance of 3 cm from the patient's elbow. However, since it is a manual placement process, an exact location in the same spot on the arm cannot be guaranteed. Finally, each session focused on a different UL to facilitate the distributed practice principle, aiming to prevent early fatigue. The first day of intervention started with the less affected hand.

### Video game description

**Fig. 1:**
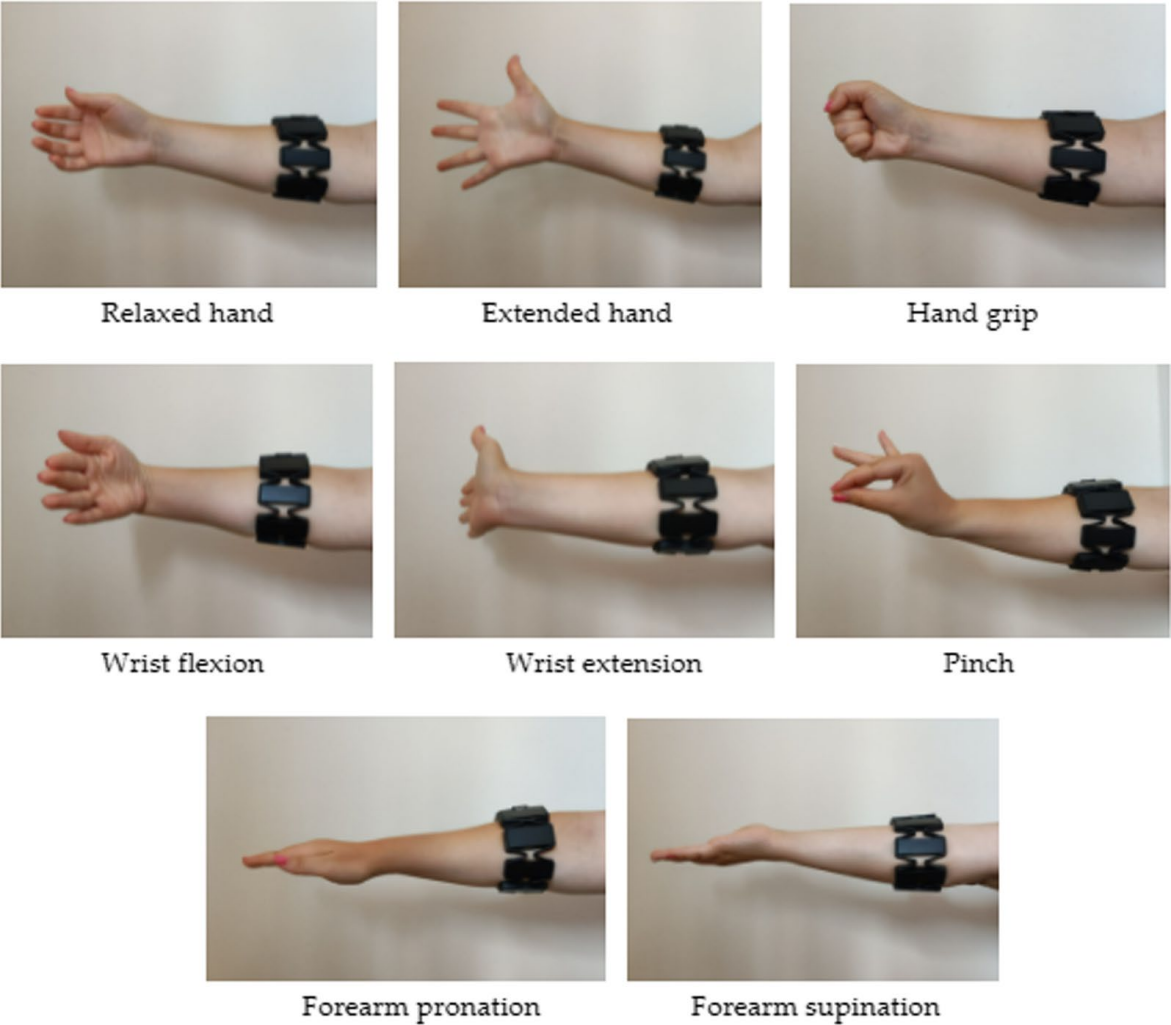
Gestures used for video game design

For this study, four specific video games were developed for the treatment of UL impairments in patients with MS. Each video game was designed to simulate the movements and exercises typically included in conventional physical therapy protocols, such as hand opening and closing, wrist flexion and extension, finger pinch and forearm pronation and supination. Thus, a total of 8 gestures, including the resting position or relaxed arm, were used in this study (Fig. 1).

The essential principle of the serious games developed for this study is to promote the repetition of specific movements in a more motivating environment, where the actions in the video game are controlled through hand gestures. The MYO Armband® sensor was used to capture sEMG activity in the forearm during the execution of training exercises and identify the gestures performed by the patient. MATLAB® software (MathWorks®, version 2020) was used to transform the sEMG signals captured by the sensor into information about wrist and hand gestures. The Unity game development engine (Unity Technologies, 2023) was used to create the different virtual environments.

Thus, it can be observed that the proposed system consists of two modules: (1) the game module and (2) the gesture recognition module. Both subsystems were implemented on different development platforms due to their particular characteristics. Consequently, a method for information transfer is required. In this case, a client–server method was implemented to communicate the games and the gesture recognition block using TCP/IP sockets, with the game being on the server side and the gesture classifier on the client side.

**Fig. 2:**
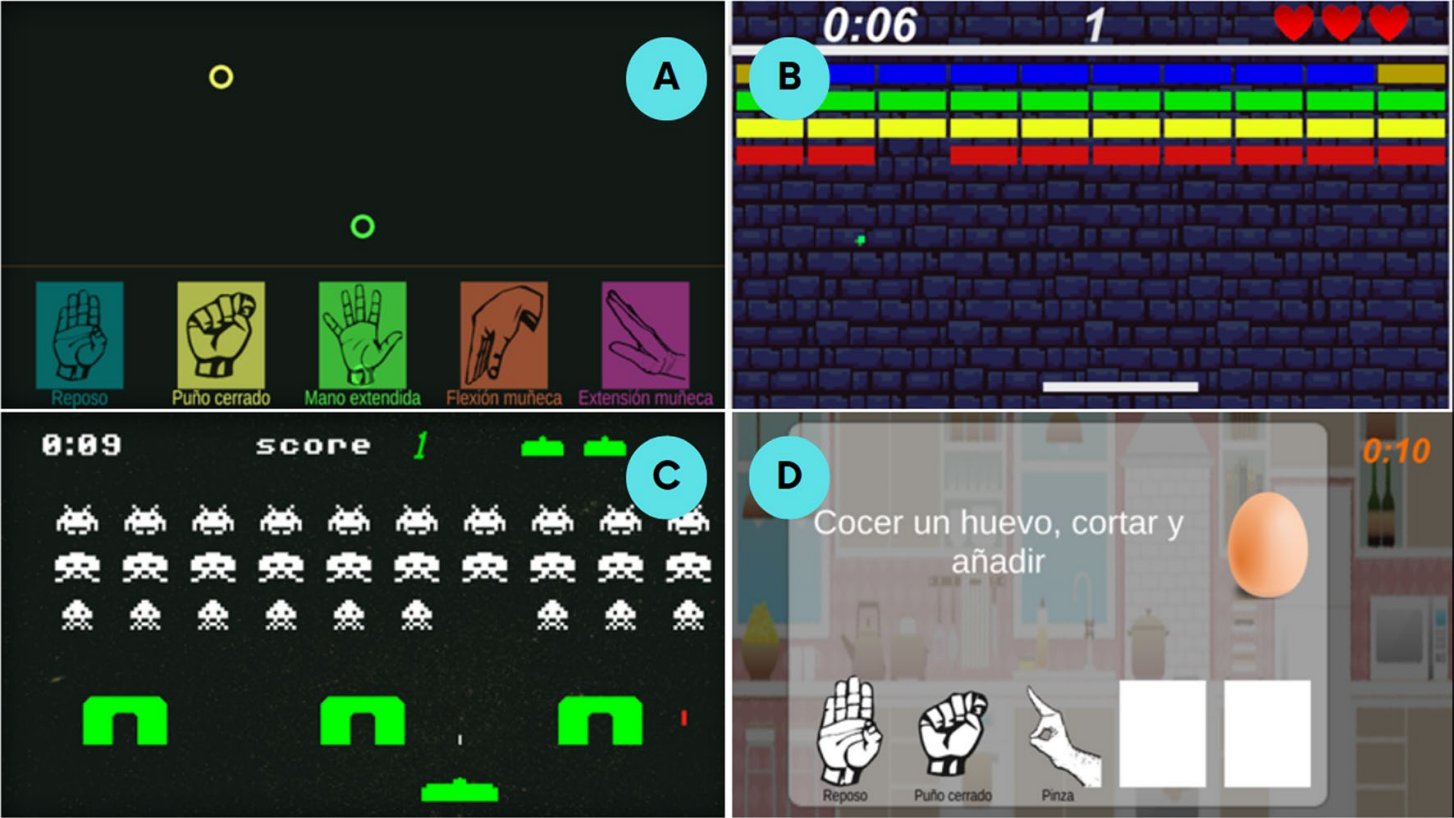
**A** MYO-Gesture. **B** MYO-Arkanoid. **C** MYO-Space. **D** MYO-Cooking

The set of video games used in this protocol were: *MYO-Gesture*, *MYO-Arkanoid*, *MYO-Space* and *MYO-Cooking* (Fig. 2). A comprehensive description of the video games was developed in a previous study [18]. However, the main features of each game are described below:

- *MYO-Gesture*: in this game, a set of coloured rings fall from the top of the screen, each associated with a random gesture that the patient has to imitate. If the gesture is performed correctly, the player earns a point and the music continues playing until the predetermined game time ends (Fig. 2A).
- *MYO-Arkanoid*: this is an arcade-style game consisting of a set of blocks that the player has to break by bouncing a ball on a movable paddle controlled by wrist movements. The paddle moves from left to right through gestures. It is controlled by wrist flexion and extension or by forearm pronation and supination movements. Each time a block is destroyed, the player earns one point, or five points in the case of a golden block. The game ends when all the blocks are destroyed or when the player loses all three lives given at the beginning of the game (Fig. 2B).
- *MYO-Space*: this game is based on the arcade game "Space Invaders" (Taito Corporation, Tokyo, Japan; Midway Games, Chicago, USA). The user controls a spaceship moving from left to right using gestures. The objective is to dodge enemy attacks while shooting to eliminate them with another gesture. The player's spaceship can pass through the barriers, unlike the enemy attacks, which gives the player an advantage. The game ends when the player eliminates all the invaders or loses all three lives given at the beginning of the game (Fig. 2C).
- *MYO-Cooking*: this game consists of following a pre-configured cooking recipe (including ingredients and steps) set by the therapists. Each step of the recipe is completed by repeating a sequence of UL movements. For example, in the case of frying an egg, the patient can add oil to a pan by performing a forearm pronation gesture, crack the eggshell with a fist gesture and add salt with a pinch gesture. When all the steps are completed, the game considers the recipe finished (Fig. 2D).

**Fig. 3:**
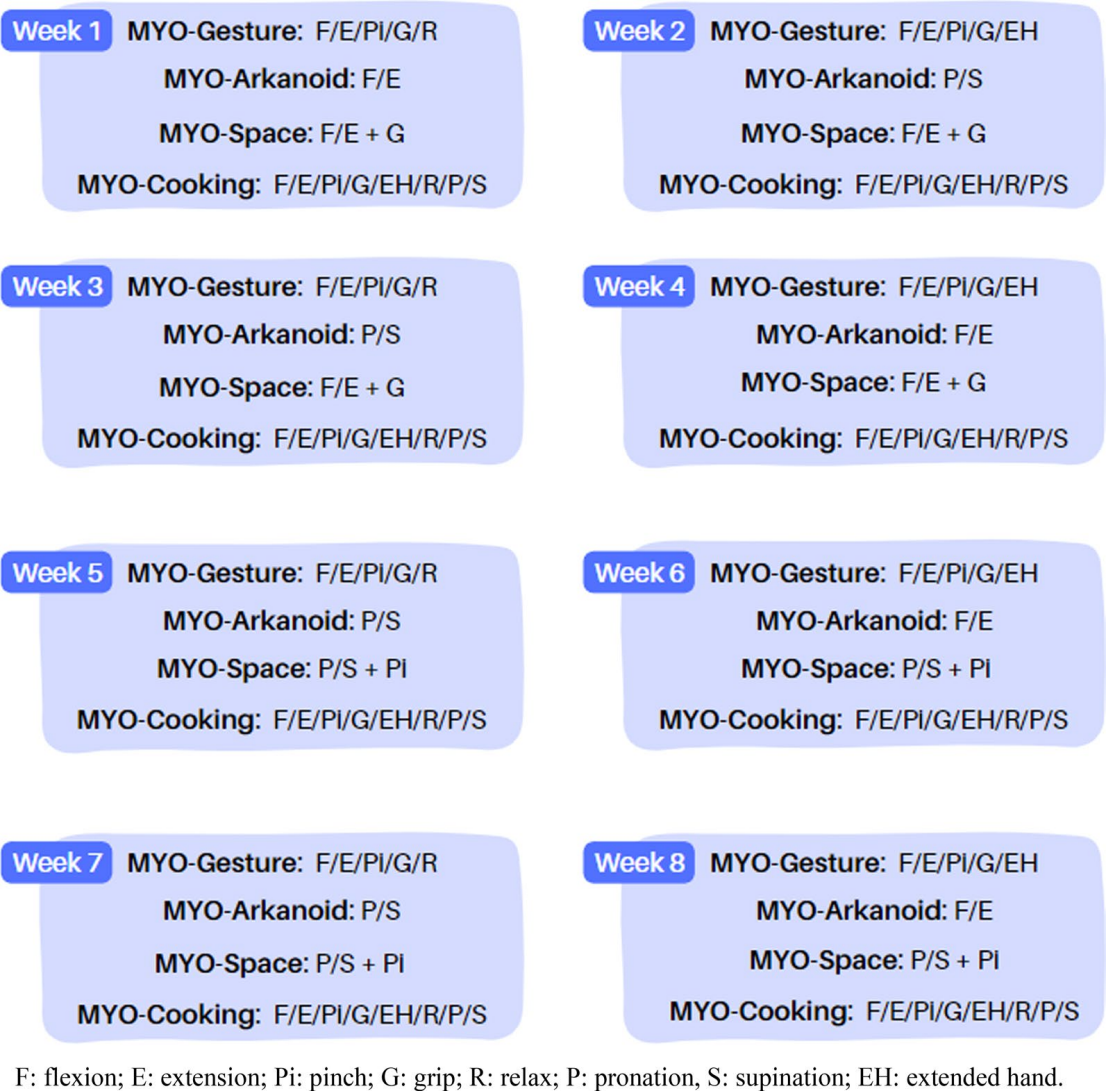
VR intervention protocol with MYO Armband®

The treatment protocol used in this study is shown in Fig. 3. In each session, the patient played each game for 3 min and 45 s.

### Outcome measures

All assessments were conducted by three trained physical therapists who were blinded to the intervention received by the participants. The following outcome measures were administered in both groups at the beginning of the intervention, at the end and in a 15-day follow-up period without receiving any treatment.

#### Active range of motion

Joint range of motion was assessed using a universal goniometer following the Norkin recommendations [25]. Data on wrist flexion and extension, forearm pronation and supination were recorded as these were the primary movements used in the experimental intervention video games.

#### Handgrip strength

Grip strength was measured using the Jamar® hydraulic hand dynamometer, which consists of a grip handle and a maximum force indicator with a dual-scale in pounds (0–198 lb) and kilograms (0–90 kg). The maximum force indicator remains after each reading until reset for easy reading. The isometric design and hydraulic system ensure highly accurate and reproducible results [26]. Each patient performed three readings on each side and the average value of the three measurements in kilograms was taken as the result, following the recommendations of Mathiowetz et al. [27], hand dynamometry has been widely used in the context of MS to assess grip strength [28–30] and is recommended by the American Society of Hand Therapists (ASSH) and the Brazilian Society of Hand Therapists [31]. It is recognised as an objective index for functional hand assessment [26].

#### Box and Block Test (BBT)

This test was used to evaluate coordination, speed of movement and unilateral gross motor skills in both UL. The test involves moving as many blocks as possible from one side to the other of a box, crossing the midline, within one minute. The score is determined by counting the number of blocks transferred from one compartment to another during that time. If multiple blocks are transferred together, only one block is counted as valid. Higher scores on this test indicate greater gross manual dexterity [32]. The BBT is a quick, simple and reliable assessment tool. Its administration and validity have been demonstrated in individuals with UL disability, including patients with MS [33].

#### Fatigue Severity Scale (FSS)

This scale consists of 9 items created by Krupp et al. [34]. The FSS evaluates the severity of fatigue and its effects on the activities and lifestyle of individuals experiencing fatigue, such as those with MS, for which it has been validated [35]. According to the established norms, the items are quantified on a 7-point scale, where "1" represents "strongly disagree" and "7" represents "strongly agree." The minimum score is 9 and the maximum is 63. Higher scores indicate greater severity of fatigue. Finally, a transformation of the score is performed into a percentage format.

#### ABILHAND

It is an outcome measure that assesses the manual abilities of adult patients. This scale measures a person's capacity to carry out daily activities that require the use of the UL [36]. Responses are coded with outcomes ranging from "impossible," "difficult," to "easy." Higher scores indicated good ability to perform ADLs that involve the use of the UL. This questionnaire is validated for individuals with MS and has excellent intra-/inter-rater reliability, excellent internal consistency and excellent convergent construct validity with the Multiple Sclerosis Impact Scale-29 (MSIS-29) [37].

#### Multiple Sclerosis Impact Scale (MSIS-29)

This scale specifically evaluates the impact of MS on the quality of life of individuals affected by the condition. It consists of 29 questions and has two dimensions: physical and psychological/cognitive. Scores range from 1 to 5, with 5 indicating a lower perceived quality of life. The maximum score in the physical part is 100 points and 45 points in the psychological/cognitive part [38, 39]. Both dimensions need to be normalised to provide the result in percentage form. Higher scores indicated greater impact of MS on the patient's quality of life. It is an easy-to-administer instrument (5–10 min) and is validated in Spanish. Furthermore, it has shown to be a valid and reliable tool compared to other assessment instruments in people with MS [40].

#### Short Symptom Questionnaire (SSQ)

This questionnaire was used to assess the possible manifestation of adverse effects resulting from the experimental treatment using the MYO Armband® sensor in the EG during the post-treatment evaluation. It includes three general dimensions: "nausea," "oculo-motor" symptoms and "disorientation." Participants were asked to rate the severity of each symptom on a five-point scale ("not at all," "mildly," "moderately," "definitely," and "severely") up to 45 min after immersion. Within these three dimensions, the SSQ includes symptoms such as fatigue, headache, blurred vision and increased salivation, among others. Higher scores indicated greater perception of adverse effects resulting from the experimental intervention. Although it has not yet been validated as an independent measure in Spanish, the SSQ provides a convenient profile of symptoms experienced with VR systems [41].

#### NASA-Taskload index

This questionnaire was used to assess the perceived workload of the EG in the post-treatment evaluation. This questionnaire has been widely used in aviation, nuclear engineering, medicine and VR applications to understand the perceived task load immediately after the task is performed through mental, emotional and physical dimensions [42, 43]. It is divided into six parts (mental demand, physical demand, temporal demand, performance, effort and frustration). Each part is analysed in a percentage value (%) and a total score is calculated. Higher scores indicated a greater perceived workload by the patient in the experimental intervention.

#### System Usability Scale (SUS)

This reliable tool was used to evaluate the usability of the VR device used in the EG. It was created to assess the performance of various devices, including hardware, software, mobile devices, websites and applications used in health research. This Likert-type questionnaire consists of 10 items with five possible responses for each item, ranging from 1 "strongly disagree" to 5 "strongly agree." It has been officially translated into Spanish and has been shown to be a valid and reliable tool [44]. It is important to note that scores need to be transformed through a simple mathematical operation to obtain a percentage ranking. Higher scores on this questionnaire indicated a greater perception of usability of the system.

#### Quebec user evaluation of satisfaction with assistive technology (QUEST 2.0)

This questionnaire was used to assess the satisfaction of the participants in the EG with the device and related services provided, including items related to device weight, safety, durability, comfort, ease of use and quality of maintenance and follow-up services. It consists of 12 satisfaction items rated on a 1–5 Likert scale. This tool has demonstrated good internal consistency and is considered an important tool for patients with MS receiving rehabilitation with the assistance of a technological device [45]. For proper analysis, the questionnaire was divided into three parts: the first 8 items to analyse satisfaction with the device, the next 4 items for satisfaction with the received service and 2 additional items to evaluate overall satisfaction with the technology used. Higher scores on this scale indicated greater perceived satisfaction with the experimental intervention among all study participants.

#### Customer Satisfaction Questionnaire (CSQ-8)

Initially derived from the CSQ-18, this questionnaire consists of 8 items that assess user satisfaction with care and treatment. It was administered to both groups (EG and CG). Each item was rated on a scale of 1 to 4, with a maximum score of 32 [46]. Finally, the scores were transformed to obtain a result in a percentage form. Higher scores on this scale indicated greater perceived satisfaction among all study participants.

#### Questionnaire on satisfaction with the technology employed

This Likert-type questionnaire was designed by the research team based on previous studies [47]. It was administered to the EG and evaluated dimensions related to satisfaction with the MYO® device and the technology used, the professional who applied it, the organisation, the transferability to ADLs and the recommendation to other patients. The items were rated on a scale of 1 to 5, with a higher score indicating greater satisfaction with the described item and a lower score indicating dissatisfaction. Subsequently, the scores were transformed using a mathematical operation to obtain a percentage value. This questionnaire provides an overall view of the patients' level of satisfaction with the device, as well as their satisfaction with the professional who conducts the therapy, thus reinforcing the data obtained from the previously described questionnaire.

Therapy adherence was recorded by tracking attendance throughout the sessions and a global adherence rate to the therapy was obtained (%).

### Statistical analysis

Statistical analysis was performed using the SPSS statistical software system (SPSS Inc., Chicago, IL; v28.0). Descriptive analysis of the qualitative data was performed using means, medians, percentages and ranges. The Saphiro-Wilk test was used to screen all data for normality of distribution. For variables with a normal distribution, a repeated measures analysis of variance (ANOVA) with post hoc Bonferroni adjustments was conducted. The between-subjects factor was set as the group parameter and the within-subjects factors included each of the measurements and the treated side (more/less affected side). For the comparison of the mean differences in both groups, comparing pre-treatment with post-treatment measurements, post-treatment with follow-up measurements and pre-treatment with follow-up measurements; and the satisfaction level of the subjects and therapy attendance rate, a mean comparison analysis was conducted using Student's t-test. Effect size was obtained for the main variables with the Partial Eta Squared, which interpretation rules are: 0.01 = small effect size; 0.06 = medium effect size; and 0.14 or higher = large effect size. The statistical analysis was performed with a confidence level of 95%, considering values with p < 0.05 as significant.

## Results

**Fig. 4:**
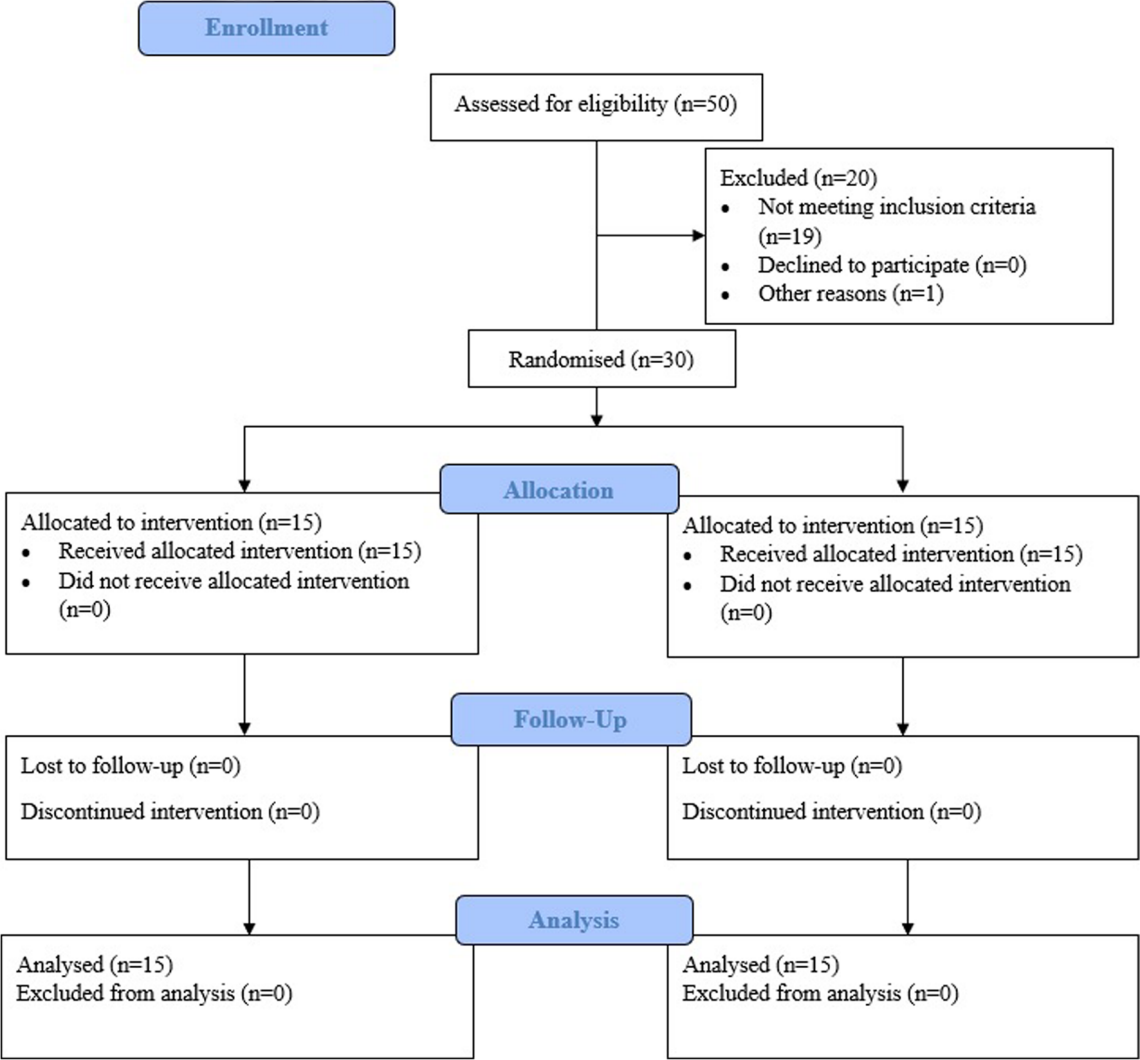
CONSORT Flow diagram

50 patients were assessed for eligibility. 19 of them were excluded from the study by not meeting inclusion criteria and 1 participant was excluded prior to the allocation due to incompatibility with the intervention protocol sessions and schedules. Finally, 30 participants, 14 male and 16 female, completed the study. Figure 4 shows a CONSORT-style flow diagram. The participants' ages ranged from 29 to 62 years (mean age 48.27 ± 7.06 years). In 14 participants, the most affected side was the left side, while the right side was the most affected for the remaining 16 participants. The type of MS was relapsing–remitting (RRMS) in 15 participants, secondary progressive (SPMS) in 10 participants and primary progressive (PPMS) in 5 participants. The duration of the disease was 15.23 ± 9.34 years. The median score on the EDSS scale was 6.0 [IQR: 1.6].

The participants were randomised into two groups, with 15 assigned to EG and 15 assigned to the CG. The sociodemographic data of the intervention groups are presented in Table 1. There were no statistically significant differences in terms of age (p = 0.762), disease duration (p = 0.239) and EDSS (p = 0.756) between the GE and the GC.

**Table 1:** Sociodemographic data of the groups

Group (n)	Age (years) Mean (± Standard deviation)	Gender	More affected side	MS type	Disease evolution (years) Mean (± Standard deviation)	EDSS Median [IQR]
Control group (15)	47.87 (± 6.7)	5 male 10 female	5 left 10 right	8 RRMS 4 SPMS 3 PPMS	13.20 (± 7.6)	6.0 [2.5]
Experimental group (15)	48.67 (± 7.63)	9 male 6 female	9 left 6 right	7 RRMS 6 SPMS 2 PPMS	17.27 (± 10.7)	6.0 [1]

*EDSS* Kurtzke Expanded Disability Status Scale; *IQR* Interquartile Range [1st quartile - 3rd quartile], *MS* Multiple Sclerosis, *PPMS* Primary-Progressive MS, *RRMS* Relapsing–Remitting MS, *SPMS* Secondary-Progressive MS. Data are expressed as mean (± Standard deviation) or median [IQR]

### Inter-group analysis

The ANOVA analysis showed statistically significant differences in active joint range of motion in the group*time comparison for wrist dorsiflexion (F = 4.737; p = 0.02) and wrist palmar flexion (F = 7.573; p = 0.003). However, the post hoc analysis did not reveal significant differences between the two groups. There were no statistically significant differences in the group*side*time comparison.

On the other hand, the ANOVA analysis showed statistically significant differences in active joint range of motion in the group*side*time comparison for forearm pronation (F = 3.515; p = 0.048) and forearm supination (F = 7.293; p = 0.004). The post hoc analysis revealed that the significant differences shown in the ANOVA for forearm pronation were due to differences when comparing the EG with the CG in pre-treatment assessment on the most affected side (MD = -2.57; F = 9.97; p = 0.004), while for forearm supination, the differences were due to differences in the comparison of the EG with the CG in post-treatment measurements on both sides (most affected side MD = 2.73; F = 8.02; p = 0.009 and least affected side MD = 5.66; F = 6.4; p = 0.019) and in the follow-up evaluation on the most affected side (MD = 4.73; F = 53.6; p < 0.001). The rest of the results related to the active joint range of motion assessment are shown in Table 2.

**Table 2:** Analysis of inter-group data on joint range of motion

	Experimental group			Control group			Experimental group vs. Control group		
Variable	Pre Mean (± s.deviation)	Post Mean (± s.deviation)	Follow-up Mean (± s.deviation)	Pre Mean (± s.deviation)	Post Mean (± s.deviation)	Follow-up Mean (± s.deviation)	Pre p-value	Post p-value	Follow-up p-value
Dorsiflexion MAS wrist	55.00 (± 11)	56.29 (± 11)	58.14 (± 10.67)	60.00 (± 15.11)	61.00 (± 14.04)	57.57 (± 12.41)	0.412	0.458	0.971
Dorsiflexion LAS wrist	55.57 (± 13.08)	55.64 (± 13.09)	57.57 (± 12.41)	57.10 (± 12.83)	61.20 (± 13)	58.60 (11.18)	0.865	0.358	0.837
Palmar flexion MAS wrist	54.71 (± 10.09)	55.29 (± 10.85)	57.64 (± 10.6)	52.40 (± 11.92)	56.00 (± 13.04)	50.70 (± 10.41)	0.547	0.956	0.125
Palmar flexion LAS wrist	53.29 (± 16.37)	54.57 (± 15.7)	50.50 (± 11.30)	53.90 (± 15.7)	53.30 (± 12.83)	57.64 (± 10.60)	0.980	0.782	0.301
Pronation MAS	86.93 (± 2.40)	89.86 (± 0.53)	89.86 (± 0.53)	89.50 (± 1.58)	89.86 (± 0.53)	89.86 (± 0.53)	0.004*	0.426	> 0.999
Pronation LAS	87.57 (± 2.53)	89.86 (± 0.53)	89.86 (± 0.53)	88.20 (± 3.36)	86.90 (± 6.54)	89.86 (± 0.53)	0.504	0.076	> 0.999
Supination MAS	86.86 (± 3.57)	89.93 (± 1.63)	90.43 (± 0.85)	88.50 (± 2.42)	87.20 (± 3.23)	85.70 (± 2.21)	0.276	0.009*	< 0.001*
Supination LAS	88.29 (± 1.64)	89.86 (± 0.95)	89.79 (± 0.70)	88.20 (± 3.36)	84.20 (± 8.70)	88.60 (± 3.77)	0.844	0.019*	0.26

*LAS* less affected side, *MAS* more affected side

P-values expressed with Bonferroni correction

*Significant at p < 0.05

Statistically significant differences were obtained in handgrip strength measured using the Jamar® dynamometer in the group*time comparison (F = 6.665, p = 0.004). Post-hoc analysis revealed that the significant differences shown in the ANOVA were due to differences in the follow-up evaluation when comparing EG with CG (MD = 9.87, 95% CI = 1.04–18.69, p = 0.03), as shown in Tables 3 and 4.

**Table 3:** Within-group and inter-group data analysis for grip strength, coordination, fatigue, functionality and quality of life variables

Variable	Group	Pre Mean (± s.deviation)	Post Mean (± s.deviation)	Follow-up Mean (± s.deviation)	ANOVA (group*time/group*side*time) *p-*value	Paired comparison		
						Pre Vs. Post *p-*value	Pre vs Follow-up *p-*value	Post vs Follow-up *p-*value
Jamar dynamometry MAS	Experimental	27.29 (± 11.53)	27.36 (± 9.26)	29.22 (± 10.98)	0.004*/0.524	> 0.999	0.235	0.064
	Control	20.04 (± 14.80)	21.79 (± 14.49)	20.89 (± 13.91)		0.458	> 0.999	0.765
Jamar dynamometry LAS	Experimental	32.12 (± 10.23)	31.97 (± 8.89)	33.67 (± 10.62)		> 0.999	0.438	0.255
	Control	22.07 (± 12.79)	24.99 (± 13.01)	22.25 (± 12.82)		0.216	> 0.999	0.024*
BBT MAS	Experimental	45.80 (± 10.78)	48.27 (± 11.03)	50.33 (± 11.19)	0.871/0.459	0.224	0.008*	0.568
	Control	35.87 (± 13.62)	37.46 (± 14.08)	41.07 (± 15.51)		0.752	0.002*	0.079
BBT LAS	Experimental	50.00 (± 10.62)	52.20 (± 12.28)	55.13 (± 11.53)		0.290	< 0.001*	0.252
	Control	39.47 (± 13.72)	40.86 (± 13.91)	43.46 (± 12.43)		0.850	0.005*	0.371
FSS	Experimental	69.31 (± 20.84)	69.42 (21.86)	73.86 (20.45)	0.493**	> 0.999	0.745	0.271
	Control	61.79 (± 29.12)	60.43 (27.21)	60.54 (28.55)		> 0.999	> 0.999	> 0.999
ABILHAND	Experimental	37.20 (± 8.08)	37.67 (± 6.44)	37.07 (± 8.14)	0.565**	> 0.999	> 0.999	> 0.999
	Control	32.64 (± 13.70)	34.00 (± 12.41)	34.86 (± 12.29)		> 0.999	0.634	> 0.999
MSIS-29 Physical impact score	Experimental	43.67 (± 24.80)	53.67 (± 16.15)	52.00 (± 27.12)	0.212**	0.06	0.331	> 0.999
	Control	47.41 (± 32.79)	47.50 (30.52)	47.77 (± 31.15)		> 0.999	> 0.999	> 0.999
MSIS-29 Psychological impact score	Experimental	36.66 (± 18.85)	44.06 (± 27.04)	42.03 (± 28.94)	0.196**	0.317	0.681	> 0.999
	Control	50.19 (± 28.06)	45.83 (± 34.40)	48.21 (± 33.18)		> 0.999	> 0.999	> 0.999

*BBT* Box and Blocks Test, *FSS* Fatigue Severity Scale, *LAS* less affected side, *MAS* more affected side, *MSIS-29* Multiple Sclerosis Impact Scale-29

P-values expressed with Bonferroni correction

*Significant at p < 0.05

**Comparison only made by group*time

**Table 4:** Inter-group data analysis for grip strength, coordination, fatigue, functionality and quality of life variables

Variable	Experimental group Mean ± SD	Control group Mean ± SD	Mean difference	*p*	CI 95% of mean difference
JAMAR pre-treatment	29.70 (± 10.66)	21.38 (± 13.37)	8.33	0.07	− 0.72 to 17.37
JAMAR post-treatment	29.66 (± 8.95)	23.39 (± 13.19)	6.27	0.14	− 2.15 to 14.71
JAMAR follow-up	31.44 (± 10.63)	21.58 (± 12.83)	9.87	0.03*	1.04 to 18.69
BBT pre-treatment	47.9 (± 10.04)	37.67 (± 13.3)	10.23	0.024*	1.42 to 19.05
BBT post-treatment	50.23 (± 11.07)	39.17 (± 13.39)	11.07	0.02*	1.81 to 20.25
BBT follow-up	52.73 (± 10.72)	42.27 (± 13.45)	11.47	0.026*	1.37 to 19.57
FSS pre-treatment	69.31 (± 20.84)	61.8 (± 29.12)	7.51	0.428	− 11.67 to 26.71
FSS post-treatment	69.42 (± 21.86)	60.43 (± 27.21)	8.99	0.334	− 9.76 to 27.73
FSS follow-up	73.86 (± 20.45)	60.54 (± 28.55)	13.32	0.158	− 5.5 to 32.14
ABILHAND pre-treatment	37.20 (± 8.08)	32.64 (± 13.71)	4.56	0.281	− 3.95 to 13.06
ABILHAND post-treatment	37.67 (± 6.44)	34.00 (± 12.41)	3.67	0.322	− 3.79 to 11.13
ABILHAND follow-up	37.07 (± 8.14)	34.86 (± 12.29)	2.21	0.57	− 5.7 to 10.1
MSIS-29 PD pre− treatment	43.67 (± 24.80)	47.41 (± 32.79)	− 3.74	0.73	− 25.8 to 18.31
MSIS-29 PD post-treatment	53.67 (± 16.15)	47.5 (± 30.52)	6.17	0.498	− 12.25 to 24.59
MSIS-29 PD follow-up	52.00 (± 27.12)	47.77 (31.15)	4.23	0.699	− 17.98 to 26.44
MSIS-29 CD pre-treatment	36.66 (± 18.85)	50.20 (± 28.06)	− 13.54	0.136	− 31.64 to 4.56
MSIS-29 CD post-treatment	44.06 (± 27.04)	45.83 (± 34.40)	− 1.77	0.879	− 25.25 to 21.72
MSIS-29 CD follow-up	42.03 (± 28.94)	48.21 (± 33.18)	− 6.18	0.597	− 29.87 to 17.5

*CD* cognitive dimension, *CI* confidence interval, *BBT* Box and Blocks Test, *FSS* Fatigue Severity Scale, *MSIS-29* Multiple Sclerosis Impact Scale-29, *p* p-value, *PD* physical dimension, *SD* standard deviation

P-values expressed with Bonferroni correction

*Significant at p < 0.05

No significant differences were found in the ANOVA for BBT by group*time or group*side*time (Table 3). However, post-hoc analysis showed significant differences in all measurements conducted for the BBT (Table 4).

For the remaining variables (FSS, ABILHAND, MSIS-29), no statistically significant differences were observed in the ANOVA analysis for the group*side, as Table 3 shows.

Effect sizes for joint range of motion, grip strength, coordination, fatigue, functionality and quality of life variables are shown in Table 5.

**Table 5:** Effect sizes for joint range of motion, grip strength, coordination, fatigue, functionality and quality of life variables

Variable	F	ANOVA p-value	Partial eta square
Wrist dorsiflexion	0.52	0.5	0.023
Wrist palmar flexion	0.01	0.93	< 0.001
Pronation	1.9	0.18	0.08
Supination	2.77	0.11	0.112
Jamar dynamometry	3.75	0.06	0.118
BBT	6.08	0.02*	0.178
FSS	1.26	0.27	0.05
ABILHAND	0.891	0.35	0.032
MSIS-29 Physical impact score	0.054	0.82	0.002
MSIS-29 Psychological impact score	0.512	0.5	0.02

*BBT* Box and Blocks Test, *FSS* Fatigue Severity Scale, *MSIS-29* Multiple Sclerosis Impact Scale-29

*Significant at p < 0.05

Satisfaction with the rehabilitation intervention in EG was 90 (± 11.88) points, while in GC it was 94.79 (± 6.54) points out of 100. There were no statistically significant differences between the two groups (p > 0.05). Satisfaction with the technology used, as evaluated in EG through the Likert-type questionnaire, showed a score of 84.36 (± 7.95) out of 100 points.

EG obtained a treatment attendance rate of 97.08% (± 5.21), while CG got an attendance rate of 91.25% (± 7.34). There were statistically significant differences in the attendance percentage between the two groups (F = 5.43, p = 0.029).

### Intra-group analysis

**Fig. 5:**
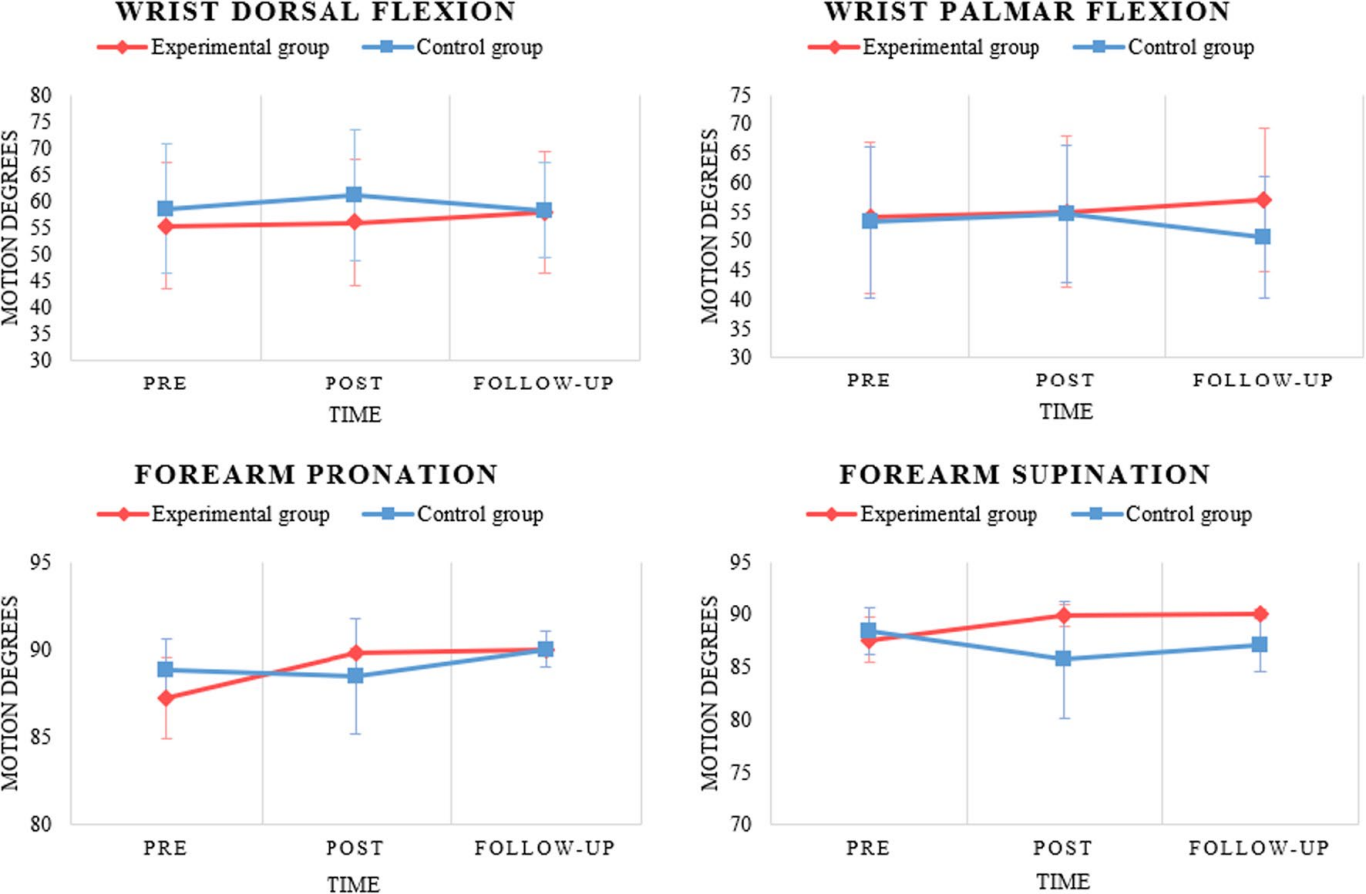
Active range of motion graphs over time and group. *LAS* less affected side; *MAS* more affected side

**Fig. 6:**
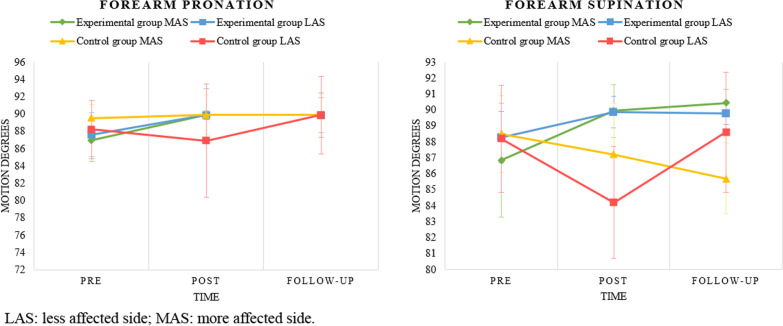
Active range of motion graphs over time, group and side

Regarding the data obtained from the three measurements of active range of motion in wrist dorsiflexion, no statistically significant changes were detected across the measurements in either group, despite slight clinical changes experienced in both EG and CG patients, which may be influenced by the Standard Error of Measurement of the goniometer evaluation. For wrist palmar flexion, statistically significant improvements were recorded in the comparison between pre-treatment and follow-up evaluation of the more affected side in the EG (p = 0.036). Statistically significant improvements were also observed in forearm pronation in the comparison between pre-treatment and post-treatment (p < 0.001) and pre-treatment and follow-up evaluation (p < 0.001) of the more affected side, as well as in the comparison between pre-treatment and follow-up evaluation (p = 0.014) of the less affected side in the EG. Regarding forearm supination, statistically significant improvements were recorded in the comparison between pre-treatment and post-treatment measurements (p = 0.03) and pre-treatment and follow-up evaluation (p = 0.003) of the more affected side in the EG, compared to a significant worsening in the data in the comparison between post-treatment and follow-up evaluation (p = 0.03) of the more affected side in the CG. Figures 5 and 6 show the data regarding the range of motion in flexion, extension, pronation and supination over time in both groups in a graph form with means and standard deviations.

No significant changes were observed in the measurement of handgrip strength using the Jamar® dynamometer in the EG, despite the clinical improvement experienced in both sides, as shown in Table 3. However, the CG recorded significant worsening changes in the comparison between post-treatment and follow-up (p = 0.024) in the less affected side.

The BBT showed statistically significant changes in both EG and CG in both UL in the comparison between pre-treatment and follow-up evaluation. These data are presented in Table 3.

No significant changes were achieved over time in the FSS, ABILHAND, or MSIS-29 scales (physical dimension and cognitive dimension).

The SSQ questionnaire showed an average score of 7 (± 10) out of 100 points. The results on perceived workload estimation by EG patients, as measured by the NASA-Task Load Index questionnaire, were 25 (± 12.83) out of 100 points.

An average score of 80.66 (± 11.47) out of 100 points was obtained in the SUS. In the QUEST 2.0 scale, scores of 34.73 (± 4.36) out of 40 points were obtained for questions related to satisfaction with the device, 16.66 (± 3.66) out of 20 points for questions related to satisfaction with the received service and 8 (± 1.85) out of 10 points for general satisfaction with the technology used.

The results obtained in the post-intervention assessment of satisfaction with the MYO® technology applied to the EG showed an average score of 84.36 (± 7.95) out of 100 points, indicating that the patients were highly satisfied with the VR MYO® intervention.

## Discussion

The purpose of this study was to investigate the effects of the MYO Armband® sEMG motion capture system, combined with a conventional physical therapy treatment program, on patients with MS. To the best of our knowledge, this is the first RCT that attempts to analyse the effects of using video games with the MYO® capture system on UL function in patients with MS.

Our results showed significant inter-group differences in active range of motion in forearm supination (p = 0.004) and manual grip strength (p = 0.004). Additionally, at the intra-group level, statistically significant improvements were observed in active range of motion in wrist flexion, forearm pronation and forearm supination in the EG. On the other hand, clinical improvement in handgrip strength was observed in the more affected side of the EG compared to the CG, whose data showed significant worsening in the follow-up evaluation compared to the post-treatment measurement. Significant intra-group improvements in manual dexterity were also observed throughout the measurements in both groups.

In recent years, several studies have been conducted on UL rehabilitation in patients with MS. Cuesta-Gómez et al. [48] investigated the effectiveness of using a set of specifically designed video games with the Leap Motion® capture sensor on strength, coordination, movement speed, fine and gross motor skills, fatigue and quality of life in patients with MS with an EDSS score between 3.5 and 7.5. The protocol consisted of a total of 20 physical therapy sessions over 10 weeks, including 15 min of VR combined with a 45-min conventional physical therapy treatment. They found effects on fine and gross manual dexterity, as well as coordination of the UL, along with high satisfaction and treatment adherence. Waliño-Paniagua et al. [49] conducted a study to analyse the effects of a 30-min conventional occupational therapy intervention combined with 20 min of VR using a webcam and online available video games. A total of 20 sessions were carried out over a 10-week period. They observed clinical improvements in the precision of several UL movements and increased effectiveness in the execution of certain functional tasks. Cuesta-Gómez et al. [50] conducted a study aiming to evaluate the effects of using the commercial video game Brain Training Dr Kawashima® with the Nintendo Switch® combined with a conventional rehabilitation intervention on grip strength, coordination, dexterity, movement speed, functionality, quality of life and executive function. A total of 16 sessions were applied over an 8-week period, consisting of 40 min of conventional occupational therapy combined with a 20-min video game therapy protocol (10 min for each side). The results were compared with a CG that received the same number of sessions of conventional occupational therapy lasting 60 min. The EG showed improvements in grip strength, coordination, fine and gross motor skills and UL functionality. No differences were observed between the two groups. Additionally, a high level of satisfaction with the therapy was reported, resulting in a high rate of therapeutic adherence. None of the groups experienced adverse effects. Jonsdottir et al. [51] conducted a clinical trial to investigate the effects of using a video game system (Rehab@Home) to enhance neurorehabilitation services for the UL of patients with MS and evaluate its clinical efficacy. Sixteen patients were included and randomised into two groups, both receiving their usual therapy. One group underwent 12 sessions of 45-min MS rehabilitation using serious games with the Kinect sensor, while the other group received the same number of sessions using commercial Nintendo Wii® games. Manual dexterity was assessed using the Nine Hole Peg Test and BBT and quality of life was measured using EQ-VAS and SF-12. Significant improvements were observed in the speed of fine pinch movements, grip and hand opening in the group that received therapy with serious games. Satisfaction with the Rehab@Home method was high.

All these findings are consistent with those observed in our study. The statistical analysis showed significant results for the variables of active range of motion and grip strength, as also shown in the work of Cuesta-Gómez et al. [50]. From our perspective, we believe that these changes observed in our study may be attributed to the specific nature of our video game protocol, which games focused primarily on concentric isotonic contraction to perform the corresponding gesture followed by an isometric contraction of the forearm muscles to generate the maximum range of motion for the corresponding gesture to interact with the virtual environment. In other words, we believe that the changes in range of motion and grip strength were a result of the specific trained tasks (and type of contractions) provided through the games in our protocol.

Regarding the results on inter-group differences observed in the pre-intervention evaluation for the forearm pronation variable, our findings indicated that the CG had a wider range of pronation mobility in the more affected side compared to the EG. However, this range of motion in the EG improved over the measurements, reaching similar results in the follow-up evaluation as the CG (Fig. 6). This indicates that the protocol proposed through the MYO Armband® sensor was able to improve the range of motion for forearm pronation in the patients included in the study. Anderton et al. [52] highlighted the importance of forearm and wrist mobility for the proper execution of functional movements of the UL, whose main purpose is to stabilise the hand and enable the ability to manipulate objects in the subjects' environment. They also emphasise the importance of the range of motion of pronation-supination movements, which is crucial for ADLs related to feeding and self-care. Therefore, the improvement data collected in terms of active range of motion of the forearm and wrist in the EG of our study indicate that the MYO Armband® system could be considered an effective tool for improving mobility in patients with MS. Nevertheless, considering the contributions of Ryu et al. [53] and Gates et al. [54] regarding the functional range of motion required for the proper performance of ADLs, the results of our study should be interpreted with caution because both groups started from an initial range of motion of the forearm and wrist that was already functional. Therefore, it might be interesting to conduct future research in patients with MS with a range of motion of the forearm and wrist below the limits of functionality to further investigate the potential effects observed in our study.

Our study found inter-group differences in terms of coordination and motor dexterity in the post-hoc analysis for all measurements as Table 4 indicates. All these differences remained from the pre-treatment evaluation until the follow-up evaluation. Additionally, the statistical test for analysing mean differences in both groups, comparing pre-treatment with post-treatment measurements, post-treatment with follow-up measurements and pre-treatment with follow-up measurements, did not show significant differences (p > 0.05). Therefore, there were no significant inter-group differences resulting from the proposed treatment for these variables in the study since the groups started from a different baseline situation for the BBT, which remained consistent throughout the study. From our perspective, this lack of differences may be due to the total treatment dosage used, which might not have been sufficient to achieve changes at this level. Furthermore, the fact that only one side was treated in each VR session could also contribute to this lack of inter-group differences. In future research, it would be beneficial to increase the treatment dosage and to allow interaction with the virtual environment using both UL through two MYO Armband® sensors (one for each arm) to achieve greater effects on coordination and manual dexterity in people with MS.

Fatigue, functional abilities of the UL or quality of life variables also did not show significant inter-group differences. This could be due to the duration of each treatment session, the overall duration of the experimental intervention and a hypothetical perceived fatigability [55] that could limit the application of more intensive intervention strategies with these patients. However, intra-group improvements were observed over time in some of the scales in both groups, such as the BBT, which is consistent with the findings in the studies by Cuesta-Gómez et al. [48, 50] and Jonsdottir et al. [51].

It should also be considered that all patients recruited for this study had a range of disability from moderate (EDSS: 3.0) to wheelchair restricted (EDSS: 7.5), so both the conventional and experimental protocols over the 8-week treatment period could contribute to maintaining stability at a clinical level for the analysed variables, which is also a positive outcome in a degenerative and progressive condition like MS.

Furthermore, a low score obtained in the SSQ and NASA-Task Load Index leads us to conclude that the proposed protocol presented as a safe therapeutic approach, with a very low incidence of adverse effects, which included feelings of fatigue, difficulty concentrating and a sense of “fullness of the head”, providing a low sense of workload burden on the patients included in the study.

Regarding the usefulness of the MYO® system as measured by the SUS, the items with the highest scores among the participants in the EG were: "*I think I would like to use this system frequently*", "*I felt the system was easy to use*," and "*I felt very confident using the system*." These data indicate the good acceptance of the system by the participants, its ease of use and the perception of safety during its utilization.

Finally, the high satisfaction data with the technology obtained concur with the results of the studies mentioned earlier [48–51], where a high rate of satisfaction with the employed technology was also reported. On one hand, the most notable items on the QUEST 2.0 scale were "*satisfaction with fit, comfort and adjustability of the device*," "*satisfaction with the quality of professional services*," and "*general satisfaction with the device and services provided*," coinciding with the most notable items on the Likert-type Scale of Satisfaction with the Technology employed, which were "*satisfaction with the proposed games*," "*satisfaction with the progression in game difficulty*," "*satisfaction with personalized attention*," and "*general satisfaction with the program*." Additionally, the statistically significant differences between the two groups in terms of therapy attendance percentage suggest that combining conventional physical therapy with the MYO® system results in greater treatment adherence and it captures better the attention of patients compared to conventional therapy alone. However, treatment adherence exceeded 90% in both groups. This may be attributed to the fact that patient recruitment was conducted in an Association where patients are highly engaged to their therapy, in a comfortable and safe environment, with professionals adequately trained to meet the therapeutic needs of the patients. Nevertheless, the EG even showed higher treatment adherence than the CG, leading us to conclude that treatment approaches based on new technologies, added to the conventional therapy, promote and increase this patient attendance.

This study has several limitations. First, the results cannot be generalised to the entire population with MS or other neurological conditions, as this research was conducted solely with patients with an EDSS score between 3.0 and 7.5 and a specific disease duration. Second, a limited total dose of treatment under MYO® system for each UL could be a potential limitation of our methodology. Third, it would be interesting to explore in future studies the effects in patients with MS with different levels of disability, disease duration, greater functional limitation of forearm and wrist range of motion and with a balanced ratio men/women in the sample. Additionally, the sampling method may have resulted in selection bias as the patients were recruited from a single MS Association in a specific location. Finally, longer follow-up evaluations would be valuable in future studies.

## Conclusion

The MYO Armband® motion capture system, combined with a conventional physical therapy program, produced effects on wrist active range of motion and handgrip strength in patients with MS with an EDSS score between 3.0 and 7.5, with high system usability, high satisfaction with the technology used and an excellent therapeutic adherence rate. Moreover, the use of the MYO® system proved to be a safe therapeutic strategy, as indicated by low scores for adverse effects and perceived workload.

## Data Availability

Data are available from the corresponding authors upon reasonable request.
